
JOURNAL INFORMATION
==============================
NLM Title Abbreviation: Alcohol Res
Journal ID: Alcohol Res
NLM Journal ID: 101594475
Journal ID: ARCR

Alcohol Research : Current Reviews

ISSN: 2168-3492
EISSN: 2169-4796
Publisher: National Institute on Alcohol Abuse and Alcoholism

ARTICLE INFORMATION
==============================
PMCID: PMC3860412
Article ID: arcr-34-3-375
Article version: 1
Subjects: Genetics Glossary

Genetics Glossary

Print publication date: 2012
Volume: 34
Issue: 3
First page: 375
Last page: 377
Copyright year: 2012
License: Unless otherwise noted in the text, all material appearing in this journal is in the public domain and may be reproduced without permission. Citation of the source is appreciated.
License URL: https://creativecommons.org/publicdomain/mark/1.0/

==============================
Agonist: An agent that mimics the actions or effects of another agent at a receptor (e.g., a drug that mimics the effects of a neurotransmitter).

Allele: One of two or more forms of a gene that reside at the same position on a pair of chromosomes; different alleles of a gene may serve the same function (e.g., code for an enzyme that breaks down alcohol) but may result in proteins with different levels of activity (e.g., rapid or slow alcohol metabolism); alternatively, an allelic variant may not produce a functional protein.

Antagonist: An agent that blocks or reverses the actions or effects of another agent at a receptor (e.g., a drug that blocks the effects of a neurotransmitter).

Astroglia: Characteristic star-shaped cells in the brain and spinal cord that are a type of glial support cell; also called astrocytes; astrocytes and other glial support cells perform many functions to support neurons and thus, normal brain functioning.

Ataxia: Inability to coordinate muscle movements.

Candidate gene: A gene that has been implicated in causing or contributing to a particular trait (e.g., a disease); can be identified by its association with the trait and by performing linkage analyses to identify regions of the genome where such genes reside.

Chemokine: Member of a subgroup of proteins (i.e., cytokines) that are released by cells and can attract nearby target cells; chemokines are involved in several disease processes, including inflammation.

Conditional inactivation: Inactivation of a gene or protein only under certain conditions (e.g., in the presence of another chemical).

Cre-driver lines: Genetically engineered lines of laboratory animals in which the expression of a certain regulatory protein (i.e., the Cre recombinase) is controlled by cell-specific regulatory elements; these animal lines allow for the expression of other studied genes only in certain tissues or under certain conditions.

Cre recombinase: A bacterial enzyme that recognizes a specific DNA sequence called a loxP site and cuts out DNA segments that are located between two loxP sequences; animals expressing Cre recombinase in the central nervous system are used to perform site-specific gene deletions in the brain.

Cytokines: A family of molecules, produced primarily by cells of the immune system, that regulate cellular interactions and other functions; some cytokines play important roles in initiating and regulating inflammation.

Embryonic stem (ES) cells: Cells derived from early-stage embryos that still retain the ability to develop into all the types of cells that make up an organism.

Endocytosis: A process by which cells absorb molecules (e.g., proteins) by engulfing them.

Endophenotype: A trait or characteristic that is not a direct symptom of the condition under investigation but has been shown to be associated with the condition and shares a genetic cause with the condition; for example, reduced initial sensitivity to some effects of alcohol has been noted in people with greater genetic risk for alcohol use disorders and may be used as an endophenotype to identify people at risk for such disorders.

Epigenetic: A change in gene function that occurs without a change in DNA sequence; epigenetic changes can alter gene expression and may be heritable.

Epigenome: The entirety of all chemical modifications that occur within a genome without changing the DNA sequence.

Epistasis: The interaction of genes; the effect of one gene is modified by the allele that exists at a different site in the genome.

Excitatory neurotransmitter: Any neurotransmitter that in the brain acts to enhance the activity of the signal-receiving neuron.

Expression quantitative trait locus (eQTL): A quantitative trait locus (QTL) that controls the expression of a candidate gene.

Functional magnetic resonance imaging (fMRI): A type of specialized MRI used to assess brain activity during mental operations by measuring changes in blood flow that result from nerve cell activity in the brain.

Gene expression: The process by which the genetic information encoded in a gene is used to direct the creation of a gene product (i.e., protein).

Gene network: A group of genes (and the products they encode) that interact with each other to influence a certain outcome or trait.

Gene targeting: A genetic technique used to exchange a specific gene (the targeted gene) within an organism with a modified version of that gene to obtain information about the function of the targeted gene.

Gene trapping: A high-throughput genetic engineering approach to randomly insert a new DNA sequence into genes across the genome; results in the expression of the inserted, “trapped” gene and the inactivation of the endogenous gene within which the insertion has occurred.

Genetical genomics: A genetic research approach that combines traditional genetic analyses and gene expression information to identify the genetic basis of gene expression (i.e., expression quantitative trait loci [eQTLs]).

Genome: The total genetic information of an organism, cell, or species.

Genotype: The genetic makeup of an organism determined by the particular combination of alleles at one or more specific locations (loci) on one or more paired chromosomes.

Haplotype: A set of closely linked genes or genetic markers present on one chromosome that tend to be inherited together.

Heterozygous: Carrying two different alleles for a particular gene.

Homozygous: Carrying two copies of the same allele for a particular gene.

Knockin mice: Genetically engineered mice in which the gene under investigation has been inserted at a particular site (locus) in the mouse’s chromosome.

Knockout mice: Genetically engineered mice in which the gene under investigation has been inactivated by replacing it with a mutated version of the gene that does not code for a functional protein.

Long-term depression: Long-lasting mechanism contributing to neuroplasticity, whereby (depending on cell type) an episode of either very strong or very weak signal transmission at a synapse leads to a decreased effectiveness of subsequent signal transmission across that synapse.

Mesocorticolimbic reward pathway: System of interconnected brain regions that includes the ventral tegmental area, nucleus accumbens, amygdala, hippocampus, and frontal cortical regions and which is thought to mediate the rewarding effects of alcohol and other drugs, use as well as natural rewards (e.g., sweets); several neurotransmitters factor prominently in this pathway, but the role of dopamine has been most widely studied.

Messenger RNA (mRNA): Key intermediary molecule generated when a gene is expressed (i.e., when the information encoded in the gene is converted into a protein product); mRNA levels for a gene are used as an indicator of how “active” the gene is (i.e., how much of the protein is likely to be produced).

Metabolite: Intermediary product generated during the break-down (i.e., metabolism) of a particular molecule.

Microarray: A microscopic chip made from glass, plastic, or other type of support material onto which a large number of minute amounts of samples (e.g., proteins or DNA) are affixed in an orderly manner for performing automated assays of protein interactions or gene expression or for genotyping.

Microglia: Type of glial cells that act as the first and main form of active immune defense in the central nervous system (i.e., brain and spinal cord); they clear debris such as dead neurons through a process called phagocytosis, in which these cells are engulfed by the microglia and then destroyed.

microRNAs (miRNAs): a class of RNAs that do not encode proteins (i.e, noncoding RNAs) and which are approximately 21 to 23 building blocks (i.e., nucleotides) in length; they are naturally produced in cells and can interact with complementary sequences on mRNA molecules, thereby interfering with the further use of these mRNAs for protein production.

Mitochondria: Membrane-enclosed structures within cells that generate most of the cells’ energy through the production of adenosine triphosphate, a molecule that provides the energy needed for many key metabolic reactions.

Monocytes: A type of white blood cells that are part of the innate immune system and which play multiple roles in immune function.

Mutagenesis: A process by which the genetic information of an organism is changed in a stable manner, resulting in a mutation that may disrupt or alter gene function.

Neuropeptide: Protein-like molecules used by neurons to communicate with each other.

Neuroplasticity: Ability of the nervous system to change and reorganize itself throughout life by forming new connections among nerve cells or altering the activities of existing nerve cells and connections; is the basis for the ability to learn throughout life; neuroplastic changes also may underlie long-term effects of alcohol or drug exposure.

Next-generation sequencing: High-throughput technique to determine the DNA sequence of entire genomes; allows investigators to determine the order (i.e., sequence) of several hundred billion DNA building blocks per week at a dramatically reduced cost compared with traditional sequencing approaches.

Oxidase: Any enzyme that increases the rate of (i.e., catalyzes) an oxidation–reduction reaction involving molecular oxygen (O2).

Oxidative stress: An imbalance between oxidants (e.g., free radicals or reactive oxygen species) and agents that can detoxify these oxidants (i.e., antioxidants) that can lead to excessive oxidation and cell damage.

Phenotype: The observable structural or functional (e.g., behavioral, physiological, biochemical) characteristics of an individual organism; each phenotype is determined to a varying degree by the genotype and environmental factors.

Polymorphism: The presence of two or more alleles of a gene or other DNA sequence at a particular locus in a population.

Protease: An enzyme that cuts (i.e., cleaves) proteins into smaller pieces (i.e., peptides); many proteases (e.g., trypsin) cleave the proteins only at specific sites characterized by a specific sequence of amino acids.

Quantitative trait: A phenotype that varies in the degree or magnitude to which it is present (e.g., sensitivity to alcohol or height) and which typically is influenced by more than one gene.

Quantitative trait locus (QTL): A DNA region that is associated with a quantitative trait and which may contain one or more of the genes contributing to variation in that trait.

Recombinant inbred (RI) mice: A strain of genetically identical animals produced by mating successive generations of sibling animals initially descended from the offspring of a cross (i.e., a recombination) between two distinct inbred strains.

RNA interference (RNAi): A RNA transcript produced in the body that does not encode a protein but which can alter the expression of other protein-coding mRNA transcripts; also refers to the process of quelling gene expression through the use of these RNA molecules.

Sequencing: Determining the order of the building blocks (i.e., nucleotides) in a DNA or RNA segment or of amino acids in a protein.

Single-nucleotide polymorphism (SNP): A DNA sequence variation at a single nucleotide between members of a species or between paired chromosomes in an individual.

Synaptic plasticity: The ability of the connection between two neurons to change in strength in response to either use or disuse of signal transmission across the site where the two neurons interact (i.e., the synapse).

Syntenic: Pertaining to synteny-the phenomenon that the gene order along chromosomes of different species often is conserved; for example, certain genes located next to each other on mouse chromosome 4 also are located next to each other (i.e., are syntenic) on human chromosome 9.

Transcript: The product of transcription—the process by which the genetic information contained in DNA is converted into an exactly complementary sequence of RNA; used synonymously with mRNA.

Transcriptome: The entirety of all transcription products (transcripts or mRNA.) present in a cell, tissue, or organism.

Whole-genome expression profiling: High-throughput analytical approach to identify genes in a cell that are expressed at a given point in time as well as the level of gene expression.

